# Effects of Chronic Exposure to Diets Containing Moldy Corn or Moldy Wheat Bran on Growth Performance, Ovarian Follicular Pool, and Oxidative Status of Gilts

**DOI:** 10.3390/toxins14060413

**Published:** 2022-06-17

**Authors:** Yong Zhuo, Pu Yang, Lun Hua, Lei Zhu, Xin Zhu, Xinfa Han, Xiaoxue Pang, Shengyu Xu, Xuemei Jiang, Yan Lin, Lianqiang Che, Zhengfeng Fang, Bin Feng, Jianping Wang, Jian Li, De Wu, Jiankui Huang, Chao Jin

**Affiliations:** 1 Animal Nutrition Institute, Sichuan Agricultural University, Chengdu 611130, China; zhuoyong@sicau.edu.cn (Y.Z.); 2019314049@stu.sicau.edu.cn (P.Y.); hualun@sicau.edu.cn (L.H.); 201905130@stu.sicau.edu.cn (L.Z.); 202000539@stu.sicau.edu.cn (X.Z.); xfhan2012@sicau.edu.cn (X.H.); pangxiaoxue_123@sina.com (X.P.); shengyuxu@sicau.edu.cn (S.X.); 71310@sicau.edu.cn (X.J.); linyan@sicau.edu.cn (Y.L.); che.lianqiang@sicau.edu.cn (L.C.); zfang@sicau.edu.cn (Z.F.); fengbin@sicau.edu.cn (B.F.); wangjianping@sicau.edu.cn (J.W.); 14109@sicau.edu.cn (J.L.); wude@sicau.edu.cn (D.W.); 2 College of Animal Science and Technology, Sichuan Agricultural University, Chengdu 611130, China; 3 Guangxi Shangda Technology, Co., Ltd., Guangxi Research Center for Nutrition and Engineering Technology of Breeding Swine, Nanning 530105, China

**Keywords:** gilts, moldy corn, moldy wheat bran, follicular development, oxidative stress

## Abstract

Background: We investigated the effect of replacing normal corn (NC) or normal wheat bran (NW) with moldy corn (MC) or moldy wheat bran (MW) on growth, ovarian follicular reserves, and oxidative status. Methods: Sixty-three Landrace × Yorkshire gilts were assigned to seven diets formulated by using MC to replace 0% (control), 25% (25% MC), 50% (50% MC), 75% (75% MC), and 100% NC (100% MC), MW to replace 100% NW (100% MW), and MC and MW to replace 100% NC and 100% NW (100% MC + MW), from postnatal day 110 to day 19 of the second estrous cycle. Results: Feeding the gilts with MC or MW induced a lower average daily gain at days 29–56 of the experiment. Age at puberty remained unchanged, but MC inclusion resulted in a linear decrease in antral follicles with diameter >3.0 mm, and control gilts had a 12.7 more large antral follicles than gilts in the 100% MC + MW treatment. MC inclusion linearly decreased the numbers of primordial follicles, growing follicles, and corpora lutea, associated with a lower anti-Müllerian hormone level in serum and 17β-estradiol level in follicular fluid. MC inclusion decreased the serum concentrations of insulin-like growth factor 1 and its mRNA levels in the liver, combined with higher malondialdehyde concentration and lower total superoxide dismutase activities in serum and liver. Conclusion: Chronic exposure to MC-containing diets caused the loss of follicles, even if levels of deoxynivalenol, zearalenone, and aflatoxin B1 were below the levels allowed by China and Europe standards.

## 1. Introduction

The annual culling rate of sows in swine production reaches ~50% worldwide, including in China [1], Europe [2], and the USA [3]. Thus, the breeding herd has to be replenished by young replacement gilts. Consequently, the successful rearing of replacement gilts is important for the overall productive performance of the breeding herds. The successful development of gilts requires not only suitable growth and accretion of body tissues but also the synchronization of sexual development (e.g., ovarian and uterine development). The recruitment of small antral follicles to form large antral follicles is essential for the attainment of puberty for gilts. Additionally, ovarian follicle reservation can determine the lifetime fertility of mammals [4] because there are no functional oogonial stem cells in ovaries to generate new oocytes in postnatal mammals [5,6]. A large loss of oocytes during the replacement phase as gilts may have both short-term and long-term negative effects on the reproductive capacity as sows [7].

In this study, we assessed the effects of mycotoxins produced by molds (fungi) on the development of gilts, as mycotoxins are estimated to contaminate over 25% of the world’s crops [8,9]. Meanwhile, mycotoxins may induce substantial impairment of ovarian follicular development. Numerous studies have investigated the effects of mycotoxins, such as deoxynivalenol (DON), zearalenone (ZEA), aflatoxin B1 (AFB1), ochratoxin A (OA), fumonisins (FUM), and T-2 toxin (T-2), on the growth and health of pigs [10,11,12,13,14]. Awareness of the negative effect of mycotoxins on replacement gilts is growing due to the particular actions of these toxins on the reproductive tract. For example, ZEA and DON belong to the trichothecene group of mycotoxins [10]. In vivo and in vitro experiments indicated that ZEA and its metabolites exert estrogenic effects, resulting in functional and morphological alterations in ovarian follicles and oocytes, and ZEA is unequivocally implicated in reproductive disorders of swine and other domestic animals [15,16,17]. However, it is unclear whether mycotoxins have dose-dependent effects on the ovarian reserves of gilts.

Corn is the most widely available feed to provide dietary energy for domestic animals, but it is usually contaminated with mycotoxins that limit its use in swine production [18]. Wheat is another ingredient commonly contaminated by molds, and mycotoxin contamination is even greater in its byproduct wheat bran [19,20]. In grains, ZEA is often found with DON or other mycotoxins as co-contaminants because they are produced by the same fungal species [10]. Compared with a single mycotoxin, multiple mycotoxins contained in naturally moldy feed have more severe toxicity due to their synergism. However, most studies of the effects of mycotoxins on the growth and development of pigs added a purified mycotoxin to the diet [13,16,21,22,23], and the hygiene standards in China and the EU for porcine diets were established according to results based on experiments conducted with purified mycotoxin. To date, the dose-dependent effects of including different levels of mycotoxin-contaminated cereal grains on the growth performance and ovarian follicle reserve in replacement gilts is poorly understood. Therefore, we conducted this study to investigate the effects of different levels of moldy corn (MC) or moldy wheat bran (MW) on the growth and ovarian follicular development in replacement gilts.

## 2. Materials and Methods

### 2.1. Animal Ethics

All experimental procedures were approved on 2 Mart 2015 by the Animal Care and Use Committee of Sichuan Agricultural University (Approval No. 20150122), and they were conducted in accordance with the National Research Council’s Guide for the Care and Use of Laboratory Animals.

### 2.2. Preparation of Normal Corn (NC) and Wheat Bran (NW)

The corn and wheat bran were purchased from northeast China where the weather is dry and crisp to ensure that the grains had a low level of mycotoxin contamination according to our experience. The normal corn (NC) and wheat bran (NW) were placed in an environment-controlled room with temperature 28–32 °C and humidity 85–90% for 1 week to prepare MC and MW. In our preliminary study, this regimen induced the production of mycotoxins. Samples of NC and NW were left in a dry and cool place to serve as the control.

### 2.3. Animals, Diet, and Treatment

Sixty-three gilts (Landrace × Yorkshire, aged 110 ± 8 days, initial body weight (BW) 48.50 ± 3.61 kg) were used in this study. The gilts were from different dams to exclude any possible maternal effects. The control diet was formulated for two feeding phases (Table 1). In phase 1 from 1 to 28 days of the experiment, the feed contained 66.7% corn and 10.0% wheat bran to provide 3.22 Mcal/kg digestible energy (DE), 15.37% crude protein, and 0.70% standardized ileal digestible (SID) lysine. In phase 2 from 29 days to the end of the experiment, the diet contained 70.7% corn and 10.0% wheat bran to provide 3.16 Mcal/kg DE, 14.22% crude protein, and 0.63% SID lysine. Other nutrients were added to meet or exceed the requirements for growing replacement gilts recommended by NRC (2012).

To create seven dietary treatments, the NC in the control diet was replaced with MC at 25% (25% MC), 50% (50% MC), 75% (75% MC), and 100% (100% MC), the NW in the control diet was 100% replaced with MW to form the 100% MW group, and NC and NW were 100% replaced with MC and MW to form the 100% MW + MC group. Gilts were randomly assigned to one of the seven dietary groups with three replicates per treatment and three gilts per replicate for days 1–28 of the experiment. The gilts then were individually housed from day 29 until the end of the experiment.

### 2.4. Analysis of Mycotoxin Contents

The formulated diets were stored at 4 °C until feeding, and feed samples were collected for storage at −20 °C until future analysis of mycotoxin contents, including DON, ZEA, AF, FUM, OA, and T-2, using high-performance liquid chromatography–tandem mass spectrometry (HPLC–MS/MS). Briefly, feed samples were sequentially extracted, and then the extracts were pooled and cleaned using a multifunction cleaning column (MycoSpinTM400, R-Biopharm Rhone, Darmstadt, Germany) before being separated by a TC-C18 chromatographic column. The MS was conducted using electrospray ionization and multiple reaction monitoring models, according to the internal standard method. The detection limit for all specimens was 0.1 μg/kg at a sample weight of 5 g. Table 2 shows the mycotoxin content of NC, NW, MC, MW, and each diet.

For days 1–28 and days 29–56 of the experiment, the gilts were provided with feed ad libitum. They then were provided with 2.5 kg/day feed from day 57 to the end of the experiment at the 19th day of the second estrous cycle. Half of each day’s feed was provided equally twice daily at 8:00 a.m. and 4:00 p.m. Water was provided ad libitum throughout the entire experiment. The environment temperature and humidity were maintained at 20–24 °C and 50–60%, respectively. Artificial light was provided from 7:00 a.m. to 7:00 p.m.

### 2.5. Detection of Pubertal Onset and Growth Performance

All gilts were exposed (but separated by a fence) to mature boars to encourage pubertal onset at day 29 of the experiment. The naturally occurring estrous was carefully checked by one experienced stockperson on the basis of the behavioral and vulvar characteristics of the gilts [24]. Age at first observed estrous was recorded, and the day of standing heat was designed as day 1 of the first estrous cycle. The BW and backfat (BF) thickness were recorded at the beginning of the experiment, on days 28, 56, and 96, and at puberty. The BF thickness was measured at 65 mm on both sides of the dorsal midline at the last rib (P_2_) using an ultrasound scanner (Renco LeanMeater). The average daily gain (ADG) for days 1–28, 29–56, and 57–96 of the experiment was calculated, and the feed intake for days 1–28, 29–56, and 57–96 was recorded to calculate the average daily feed intake (ADFI). The feed/gain (F/G) ratio for each respective period was also calculated. The ADFI, ADG, and F/G ratio for days 1–28 of the experiment are presented for each pen as a unit; the individual gilt was the experimental unit for all parameters analyzed from day 29 to the end of the experiment.

### 2.6. Sample Collection

Blood samples before the morning meal and at 60 and 120 min after the morning meal were collected on day 18 of the second estrous cycle to measure follicle-stimulating hormone (FSH) and luteinizing hormone (LH) levels. Blood samples were also collected at day 19 of the second estrous cycle at 8:00 a.m. before the morning meal. Blood samples were allowed to coagulate for 40 min before centrifugation (3500× *g*, 10 min, 4 °C).

After serum collection, seven gilts from the control group and six gilts per group from the remaining treatment groups were killed in compliance with euthanasia principles, and the caudolateral portion of the lateral lobe of the liver was removed, snap-frozen in liquid nitrogen, and stored in a freezer at −80 °C until analysis. Ovary, uterus, and oviduct samples were washed with ice-cooled phosphate-buffered saline and dried with sterile tissue paper, and the uterus weight and length in both directions (left and right) were measured. The numbers of corpora lutea and antral follicles with diameters between 1 and 3 mm and >3 mm were recorded. The cumulus oocyte complex (COC) and granulosa cells (GCs) were obtained in large antral follicles from ovaries on both sides with diameters > 3 mm as previously described [25], snap-frozen, and stored at −80 °C.

**Figure 1:**
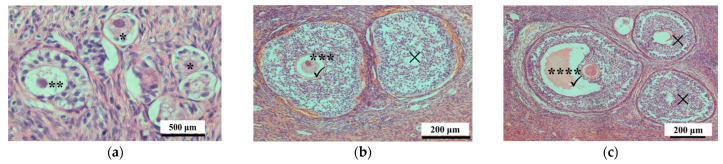
Follicle classifications: * denotes a primordial follicle and ** denotes a primary follicle in (**a**); *** denotes a secondary follicle in (**b**); **** denotes an early antral follicle with diameter <1 mm in (**c**). For primordial, primary, and secondary follicles, only those with a visible nucleus were counted (✓); those without a nucleus were not counted (×). For the antral follicles that were not easy to find in ovarian sections, those with visible oocytes were counted (✓) even they did not have a visible nucleus.

For analysis, the right ovary was cut into four sagittal slices that were then fixed in 4% paraformaldehyde (100 mmol/L phosphate buffer, pH 7.4), dehydrated, embedded in paraffin, and sectioned (5 μm thickness). One section in each part of the ovary was stained with hematoxylin and eosin (HE), and then examined under a microscope (Nikon 80i, Tokyo, Japan) for the presence of different classes of follicles, including primordial follicles, primary follicles, secondary follicles, and antral follicles; their numbers were quantified as previously described [25]. The primordial, primary, and secondary follicles with visible nuclei were counted, and those without nuclei were not counted. However, antral follicles with diameters <1 mm, which were not easy to find in ovarian sections, were counted when present in visible oocytes even if they did not have a visible nucleus (Figure 1) [25].

**Figure 2:**
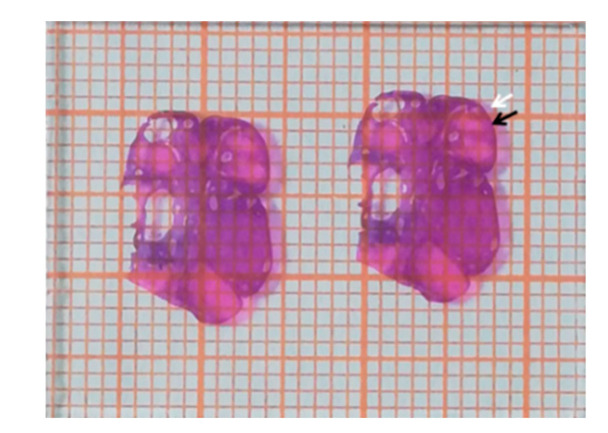
Determination of area of ovarian hematoxylin and eosin stained (HE) sections. The HE-stained sections were placed on millimeter graph paper, and the area was estimated by calculating the number of blocks covered by the HE sections. The blocks were not counted if the border of the HE sections did not occupy at least half of the block, as indicated by the white arrow. Otherwise, they were counted, as indicated by the black arrow.

The HE-stained sections were placed on millimeter graph paper, and the area was estimated by calculating the number of paper blocks that were covered by the HE sections (Figure 2). Blocks were not included if the border of the HE section did not occupy at least half of the block. The number of follicles at each stage was normalized by the area of HE-stained ovarian tissue in the sections, and results are presented as the number of follicles per cm^2^ [25]. The numbers of follicles in different parts from each ovary were pooled to calculate an average. Follicular fluid from large antral follicles was collected as previously described [26] and stored at −20 °C until analysis.

### 2.7. Analysis of Hormone Levels in Serum and Follicular Fluid

The concentrations of insulin-like growth factor-1 (IGF-1), 17β-estradiol (E_2_), and anti-Müllerian hormone (AMH) in serum or follicular fluid were measured using enzyme-linked immunosorbent assay (ELISA) kits purchased from Alpco Diagnostics (Salem, NH, USA), R&D systems (Minneapolis, MN, USA), and CUSABIO Biotech (Wuhan, China), respectively, according to the manufacturer’s instructions. The optical density (OD) value was read on an ELISA plate reader, and sample values were calculated from a standard curve according to the manufacturer’s instructions. The follicular fluid was diluted at 1:1000 for the E_2_ measurement. The detection limits of IGF-1, E_2_, and AMH were 0.09 ng/mL, 1.1 pg/mL, and 1.25 ng/mL, respectively.

### 2.8. Analysis of Oxidative Stress Biomarkers in Serum, Follicular Fluid, and Liver Samples

The thawed serum and follicular fluid samples were centrifuged at 2000× *g* at 4 °C for 20 min before the assays were conducted. Liver samples were defrosted and homogenized on ice with 0.9% NaCl. The homogenate was centrifuged at 4000× *g* at 4 °C for 20 min to obtain the supernatant for biochemical analysis. Malondialdehyde (MDA) content and enzymatic activities of superoxide dismutase (SOD) were measured in serum samples, liver homogenate, and follicular fluid samples using commercial assay kits (Nanjing Jiancheng Institute, Jiangsu, China). Briefly, MDA was quantified using the thiobarbituric acid method. The SOD activities of serum, follicular fluid, and liver homogenate samples were measured [27]. Protein concentration of liver tissues was determined [27].

### 2.9. Total RNA Extraction and Quantitative Real-Time PCR (qRT-PCR)

The total RNA of frozen liver samples and COCs was extracted using TRIzol (catalogue no. 15 596-026, Invitrogen, Waltham, MA, USA) according to the manufacturer’s instructions. The cDNA was then synthesized using a reverse transcription kit (TAKARA, Shiga, Japan) following the manufacturer’s instructions. The qRT-PCR for *IGF1*, insulin-like growth factor acid-labile subunit (*ALS*), *AMH*, growth differentiation factor 9 (*GDF9*), bone morphogenetic protein (*BMP15*), phosphatase and tensin homolog deleted on chromosome ten (*PTEN*), LIM homeobox gene (*LHX8*), cytochrome P450 family 11 subfamily A member 1 (*CYP11A1*), steroid acute regulatory protein (*StAR*), and *β-actin* was performed on a CFX96 Real-Time PCR Detection System (Bio-Rad, Hercules, CA, USA) with a commercial SYBR Green kit (TAKARA). The sequences of the primers were as follows: *IGF1*, forward 5′-ATCTGAGGAGGCTGGAGATGTA-3′ and reverse 5′-TGTACTTCCTTCTGAGCCTTGG-3′; *ALS*, forward 5′-GCTCAATGACAACCAGATCCAG-3′ and reverse 5′-CAGACAAGTTCATGACGGCCA-3′; *AMH*, forward 5′-GCGAACTTAGCGTGGACCTG-3′ and reverse 5′-CTTGGCAGTTGTTGGCTTGATATG-3′; *GDF9*, forward 5′-GGTATGGCTCTCCGGTTCACAC-3′ and reverse 5′-CTTGGCAGGTACGCAGGATGG-3′; *BMP15*, forward 5′-AGCTTCCACCAACTGGGTTGG-3′ and reverse 5′-TCATCTGCATGTACAGGGCTG-3′; *PTEN*, forward 5′-TAAAGCTGGAAAGGGACGAAC-3′ and reverse 5′-GCCTCTGACTGGGAATAGTTACTC-3′; *LHX8*, forward 5′-CTGTGCTGGCATGTTCGGT-3′ and reverse 5′-GGGCACCTTCAACACTTATTCC-3′; *CYP11A1*, forward 5′-GGCTCCAGAGGCCATAAAGA-3′ and reverse 5′-ACTCAAAGGCGAAGCGAAAC-3′; *StAR*, forward 5′-GACTTTGTGAGTGTCGGCTGTA-3′ and reverse 5′-ATCCCTTGAGGTCAATGCTG-3′; *β-ACTIN*, forward 5′-CCAGCACGATGAAGATCAAGA-3′ and reverse 5-AATGCAACTAACAGTCCGCCTA-3′. The housekeeping gene *β-actin* was amplified for each sample and used as the internal control to calculate the relative level of target gene expression using the 2^−ΔΔCt^ method.

### 2.10. Statistical Analysis

One gilt in the control, two in 25% MC, two in 50% MC, two in 75% MC, three in 100% MC, three in 100% MW, and two in 100% MW + MC did not show estrous until day 260 of age, and their data were excluded, except for their growth performance results. The growth data for days 1–28 of the experiment were analyzed using pen (replicate) as the experimental unit. The individual gilt was the experimental unit for all parameters analyzed from day 29 to the end of the experiment. The current data were checked their homogeneity of variances and normal distribution of the residuals before using parametric analyses. The data were tested for normal distribution using the Kolmogorov–Smirnov test. The data were analyzed using the general linear model procedure of SAS 9.4 (SAS Institute, Inc., Cary, NC, USA) according to the following model:

Y_i_ = µ + α_i_ + ε_i_, (1)

where Y_i_ is the response variable, µ is the overall mean, α_i_ is the fixed effect of dietary treatment, and ε_i_ is the residual error.

We used orthogonal linear contrast analysis to test the linear and quadratic effect of MC inclusion levels on parameters. Furthermore, the following statistical model was used to analyze the inclusion of MC or MW on the parameters within the control, 100% MC, 100% MW, and 100% MC + MW groups:

Y_ijk_ = μ + α_i_ + β_j_ + αβ_ij_ + e_ij_, (2)

where Y_ijk_ is the response variable, μ is the overall mean, α_i_ and β_j_ are the fixed effects of 100% MC inclusion level and 100% MW inclusion level, respectively, αβ_ij_ is the interaction among fixed effects, and e_ij_ is the residual error. The results are expressed as the mean ± pooled standard error of the mean. A *p*-value < 0.05 was considered to be statistically significant, whereas a *p*-value < 0.10 was considered to indicate a tendency.

## 3. Results

### 3.1. Effects of MC or MW Inclusion on the Growth Performance of Gilts

Table 3 summarizes the effects of the diets containing various proportions of MC or MW on growth performance of gilts. The BWs at days 1, 28, 56, and 96 of the experiment were not affected by dietary treatment, with only a tendency for 100% MC inclusion to decrease the BW at days 56 (*p* = 0.067) and 96 (*p* = 0.099) of experiment. A tendency for the ADFI of gilts to be affected by dietary treatment was detected at days 29–56 of the experiment (*p* = 0.098). The ADG of gilts at days 1–28 was affected by the inclusion of 100% MW (*p* < 0.05), and it showed linear (*p* = 0.073) and quadratic effects (*p* < 0.05) for MC. The F/G ratio at days 29–56 of the experiment was also affected by dietary treatment. A quadratic effect, but not a linear effect, of MC inclusion level on the F/G was observed at days 29–56 (*p* < 0.05). The F/G ratio at days 29–56 was also affected by inclusion of 100% MC (*p* < 0.05) or 100% MW (*p* = 0.072).

### 3.2. Effects of MC or MW Inclusion on Pubertal Onset of Gilts

Age, BW, and BF at puberty and estrous cycle length were not affected by dietary treatment (*p* > 0.05), with the exception of a tendency for BF at puberty to be affected by dietary treatments (*p* = 0.089) (Table 4). The age at puberty was affected by the quadratic effects of MC inclusion level (*p* < 0.05).

### 3.3. Effects of MC or MW Inclusion on Organ and Ovarian Follicular Development of Gilts

Table 5 lists the organ weight at slaughter on day 19 of the second estrous cycle. The weight and relative weight of the liver, spleen, and uterus were not affected by dietary treatment (*p* > 0.05). The ovary weight was affected by dietary treatment (*p* < 0.05), and a linear effect of MC inclusion level was observed (*p* < 0.05). The ovary weight of control gilts was greater than those of 100% MC and 100% MW + MC gilts (*p* < 0.05). The weight (*p* < 0.01) and relative weight (*p* = 0.074) of the ovary were decreased by 100% MC but not by 100% MW or the interaction between 100% MC and 100% MW (*p* > 0.05).

The number of small antral follicles with diameters of 1–3 mm on the ovarian surface was not affected by dietary treatment (*p* > 0.05). In contrast, the number of large follicles (>3 mm) was significantly affected by dietary treatment (*p* < 0.01) and decreased linearly with increasing MC inclusion level (*p* < 0.01). Inclusion of 100% MC or 100% MW resulted in a decrease in large antral follicles; the number decreased from 20.2 in control samples to 7.5 in 100% MW + MC gilts (*p* < 0.05). The numbers of primordial follicles (*p* < 0.01), growing follicles (*p* = 0.073), and total primordial and growing follicles (*p* < 0.01) per cm^2^ of ovarian sections were affected by dietary treatment, and a linear effect of MC inclusion level was detected (*p* < 0.01). The primordial follicles (*p* < 0.01), growing follicles (*p* < 0.05), and total primordial and growing follicles were affected by 100% MC inclusion (*p* < 0.05) but not by 100% MW inclusion or their interaction (*p* > 0.05). The number of corpora lutea was affected by dietary treatment, with a linear effect of MC inclusion level. The control gilts had 6.6 and 7.1 more corpora lutea than the 100% MC and 100% MW + MC groups, respectively (*p* < 0.05).

### 3.4. Effects of MC or MW Inclusion on the Oxidative Status of Serum, Liver, and Follicular Fluid

The antioxidant status-related parameters MDA and SOD in serum, liver, and follicular fluid were significantly affected by dietary treatment (*p* < 0.05 or *p* < 0.01), with the exception of MDA in follicular fluid (Table 6). MDA contents linearly increased, and SOD contents linearly decreased with increasing MC inclusion level in serum, liver, and follicular fluid. SOD content in liver samples showed a quadratic effect of MC inclusion level (*p* < 0.05). A higher MDA content and lower SOD content in serum, liver, and follicular fluid were detected in the 100% MC group (*p* < 0.05 or *p* = 0.055) but not in the 100% MW or the 100% MC + MW groups (*p* > 0.05).

### 3.5. Effects of MC or MW Inclusion on the Hormone Levels in Serum and Follicular Fluid

The serum levels of FSH and LH on day 18 of the second estrous cycle were not affected by dietary treatment before feeding (0 min) or at 60 and 120 min after feeding (*p* > 0.05) (Table 7). Serum IGF-1 concentration on day 19 of the second estrous cycle was significantly affected by dietary treatment (*p* < 0.01), and effects of 100% MC, 100% MW, and their interaction were detected (*p* < 0.01). The control gilts had a higher level of serum IGF-1 than the 75% MC and 100% MW groups (*p* < 0.05), while 100% MW + MC gilts had a lower serum IGF-1 level than the gilts in the other groups (*p* < 0.05). Serum E_2_ concentration was not affected by dietary treatment (*p* > 0.05), but serum AMH concentration was significantly affected by dietary treatment, with a linear effect of MC inclusion level (*p* < 0.01). Serum AMH level was also affected by 100% MC (*p* < 0.01) but not by 100% MW or the interaction between 100% MC and 100% MW (*p* > 0.05). The E_2_ concentration in follicular fluid decreased linearly with increasing MC inclusion level, and it was affected by 100% MC, 100% MW, and their interaction (*p* < 0.01). The highest level of follicular fluid E_2_ was detected in control gilts, and the lowest level was found in 100% MW + MC gilts.

### 3.6. Effects of MC or MW Inclusion on the mRNA Expression

**Figure 3:**
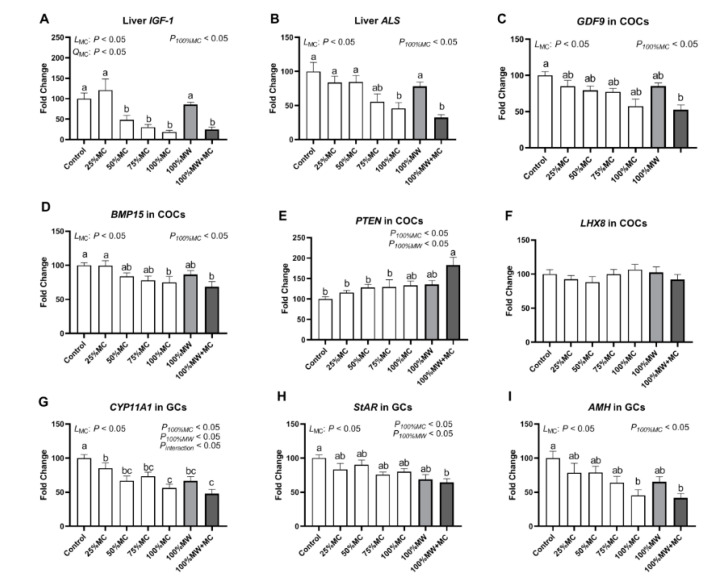
Relative mRNA expressions levels of IGF-1 (**A**) and ALS (**B**) in liver, GDF9 (**C**), BMP15 (**D**), PTEN (**E**), and LHX8 (**F**) in COCs, and CYP11A1 (**G**), StAR (**H**), and AMH (**I**) in GCs from gilts sampled on day 19 of the second estrous cycle. MC, moldy corn; MW, moldy wheat bran. L_MC_, linear response of MC inclusion; Q_MC_, quadratic response of MC inclusion. *p*_100%MC_, *p*_100%MW_, and *p*_interaction_, significance when comparing the four groups (control, 100% MC, 100% MW, and 100% MW + MC) using a mixed analysis model. COCs, cumulus oocyte complexes; GCs, granulosa cells. Different letters in columns denote differences at *p* < 0.05 (*n* = 6–7 per group).

The mRNA expression levels of IGF1 and ALS in the liver, GDF9, BMP15, PTEN, and LHX8 in COCs, and StAR, CYP11A1, and AMH in GCs were affected by the dietary treatment (*p* < 0.05) (Figure 3A–I). There were linear effects of MC inclusion level on the mRNA expression levels of liver IGF1 and ALS, COC GDF9 and BMP15, and GC StAR, CYP11A1, and AMH (*p* < 0.05). Comparison of the inclusion of 100% MC or 100% MW in a 2 × 2 set showed that the mRNA expression levels of liver IGF1 and ALS, COC GDF9, BMP15, and PTEN, and GC StAR, CYP11A1, and AMH were affected by 100% MC (*p* < 0.01 or *p* < 0.05). The mRNA expression levels of PTEN in COCs and CYP11A1 and StAR in GCs were affected by 100% MW (*p* < 0.05). The CYP11A1 mRNA level in GCs was affected by the interaction between 100% MC and 100% MW (*p* < 0.05).

## 4. Discussion

Mycotoxins, which are fungal secondary metabolites, cause serious problems for the health of domestic animals. Pigs are known to be particularly impacted by mycotoxins [28]. However, despite the hundreds of existing mycotoxins, only a small number of them are relevant to the swine production industry because they are commonly detected in cereal grains such as corn and wheat, and they are highly resistant to processing. These toxins include AF (produced by *Aspergillus flavus*) and DON and ZEA (produced by *Fusarium graminearum*) [10,28]. Growth and feed intake reduction are often the initial and most prominent symptoms observed as a result of mycotoxin consumption. Swamy et al. (2003) reported that feed intake and cumulative weight gain of starter pigs decreased linearly with the inclusion of grains naturally contaminated with *Fusarium* mycotoxins in the diet. In another pig feeding study, diets containing both AF and DON at levels >60 and 300 μg/kg, respectively, reduced growth and decreased feed intake [29]. Although the high doses of mycotoxins that induced feed refusal of pigs led to the early premise that weight gain reduction is exclusively caused by reduced food consumption, we found that the ADFI at days 1–28 and 29–56 of the experiment was not different among the different dietary groups. However, the ADG for days 29–56 of the experiment was significantly greater in the control gilts than in gilts in the 25% MC, 50% MC, 100% MC, and 100% MC + MW groups. Amuzie and colleagues offered new insight into the mechanisms contributing to growth inhibition by DON in an animal model [30,31]. They showed that DON suppressed growth in mice by reducing growth hormone (GH) signaling through mechanisms mediated by IGF-1 and ALS. Regulation of growth is complex, and the interrelated roles of ALS, IGF-1, insulin-like growth factor-binding protein 3 (IGFBP3), and GH have been thoroughly reviewed elsewhere [32]. In our study, the liver expression levels of IGF-1 and ALS and the serum levels of IGF-1 were downregulated with increasing level of mycotoxins induced by the inclusion of MC or MW, which might explain the observed decreased growth performance in gilts.

However, despite the greater mycotoxin content, the 75% MC and 100% MC diets did not result in a decreased ADG at days 29–56 compared with the control gilts. A possible explanation for this result was that gilts in these groups had a relatively lower ADG (664.6 and 674.3 g/day, respectively) at 1–28 days, but they developed adaptive physiological changes to the mycotoxins that allowed catch-up growth during days 29–56 of the experiment. Similarly, pigs were able to develop adaptation to the effects of DON-contaminated diets during an extended exposure period [11,33]. This might explain why the ADG and F/G ratio were similar between different groups at days 57–96 of the experiment.

Puberty onset is an important signature of female maturation that allows gilts to enter the reproductive cycle. It was reported that prepubescent exposure to dietary ZEA at the level of 1.5–2.0 ppm disturbed the hypothalamic–hypophysial function and stimulated an early attainment of puberty onset in gilts [8,9,10]. In the current study, we did not observe an obvious effect of MC or MW inclusion on the age at puberty, BW at puberty, or BF thickness at puberty, and this observation was consistent with early studies in which prepubescent gilts were fed with 3.61 ppm or 4.33 ppm SEN [34]. The onset of puberty is a quite complex physiological process that requires the coordination of the hypothalamus, pituitary, and ovary, and one of the most important features is the pulsatile release of FSH and LH. In the present study, we measured the serum concentrations of FSH and LH at 0, 60, and 120 min after feeding on day 18 of the second estrous cycle. We did not find any difference among groups, which suggested that mycotoxins did not directly influence the hypothalamus.

After sacrificing the gilts on day 19 of the second estrous cycle, we found that liver, kidney, spleen, and uterus development was similar among groups. Recent studies of growing pigs that were fed different levels of DON [13] and growing gilts fed with different levels of ZEA [21] also found no effects on liver development. However, the organ health (such as liver) of pigs was impaired by mycotoxins [11,13,21,22]. In the present study, we measured the antioxidant capacity in the serum, liver, and follicular fluid of gilts and found that inclusion of either MC or MW resulted in a significant elevation of MDA and suppressed secretions of SOD, suggesting that oxidative stress was induced by a high level of mycotoxins. Oxidative stress is a phenomenon that occurs in a cell when the concentration of reactive oxygen species exceeds the antioxidant capacity, and it usually impairs the reproductive performance of sows [23]. Luderer et al. suggested that oxidative stress was involved in reproductive toxicity caused by various stimuli such as a dietary toxicity. For example, T-2 toxin induced apoptosis in rat GCs through oxidative stress [35]. Researchers reported that the early oocyte environment in the follicular fluid was rich in antioxidant enzymes, which helped protect oocytes from oxidative damage [36,37]. However, the concentration of MDA and activity of SOD in follicular fluid did not differ significantly among the treatment groups in our study. However, the SOD activity in follicular fluid decreased linearly with increasing inclusion of MC or MW. SOD is an important intracellular antioxidant enzyme that detoxifies superoxide anion and provides significant protection against the oxidative effect of mycotoxin-contaminated maize [38]. In the present study, the increased oxidative stress would have consequently resulted in negative effects on ovarian development.

We observed a dose-dependent decline in large antral ovarian follicles with increasing level of mycotoxins in the diet. The number of large antral follicles decreased from 20.2 in the control group to 7.5 in the 100% MW + MC group, but the number of small antral follicles with diameter 1–3 mm remained unchanged. The decrease in large antral follicles might be due to the failure of small antral follicles to be recruited by FSH and LH. Furthermore, we found that the numbers of primary follicles and growing follicles were significantly decreased by the inclusion of MC or MW. The primary follicles and growing follicles serve as the main source of the oocyte pool, and the observed decline in the ovarian pool suggested low reproductive potential in the long-term. Recently, Zhuo et al. (2019) reported that increased follicle death during the early period of life would decrease lifetime fertility in mice. AMH is a glycoprotein produced by the GCs in growing ovarian follicles, and it serves as a biomarker of ovarian reservation [39,40]. The AMH level in gilts can also be a predictor of their subsequent fertility as sows [41]. In line with the greater size of the ovarian pool, the serum levels of AMH decreased with increasing mycotoxin levels in the diet, suggesting that greater mycotoxin content in the diet impaired ovarian function.

Ovarian follicle health and survival are regulated by signaling pathways that govern primordial follicle recruitment and growth. Reddy et al. (2008) showed that PTEN, a major negative regulator of phosphatidylinositol 3-kinase, is a critical regulator controlling oocyte growth and survival. In the present study, the mRNA expression levels of PTEN were elevated with increasing mycotoxin content in the diet, and there is evidence that mycotoxins such as FUM B1 [42], OA [43], and DON [44] can regulate PTEN signaling. Results of these studies suggest that mycotoxins might exert their negative effects on follicle survival via PTEN signaling.

We found that mycotoxins not only reduced the number of oocytes in the ovary, but also had negative effects on the oocyte developmental potential. The expression levels of BMP15 and GDF9, two well-known markers of oocyte developmental competence in mammals [45], were significantly downregulated by the inclusion of mycotoxins in the diet. Consistent with this, ZEA exposure in vitro impaired organelle function during porcine oocyte meiotic maturation [17]. Secretion of E_2_ by GCs is also important for porcine oocyte developmental competence [46]. In the present study, the concentrations of E_2_ in the follicular fluid, as well as expression of CYP11A1 and StAR, two genes encoding enzymes responsible for the de novo synthesis of E_2_, were decreased by the inclusion of MC or MW in the diet. This result suggested that mycotoxins might affect E_2_ signaling, thereby impairing porcine oocyte quality.

The current study did not provide solutions to alleviate the negative effect of mycotoxins on the ovarian follicular development. However, dietary baicalin [47,48,49] or berberine [50] supplementation could alleviate the DON challenge-induced gut impairment by decreasing oxidative stress and immune stress in pigs. It remains uncertain whether the negative effect of mycotoxins on the ovarian follicular development could be attenuated by an oxidative stress modulator.

## 5. Conclusions

In conclusion, we found that chronic exposure to diets containing mycotoxins by inclusion of MC or MW reduced the ovarian follicle pool size and oocyte quality, and these changes were associated with increased oxidative stress and suppressed liver IGF-1 and ALS secretion. According to hygienic standards in China and EU, mycotoxin levels (DON, ZEA, and AFB1) in the diet of the 75% MC group were below the maximum levels allowed for porcine diets, but this diet resulted in a significant decrease in the ovarian follicle pool and oocyte quality. Our results suggest that mycotoxin levels allowed for the production of replacement gilts should be lower than the current standards.

## Figures and Tables

**Table 1:** Diet composition and nutrient content (%).

Ingredients, %	Experimental Phase	
	Days 1–28 of Experiment	Day 29 to End of Experiment
Corn	66.70	70.70
Wheat bran	10.00	10.00
Soybean meal, CP43%	20.00	16.60
Soybean oil	1.00	-
Limestone	0.92	1.00
Dicalcium phosphate	0.27	0.60
Feed-grade sodium chloride	0.30	0.42
Choline chloride, 50%	0.10	0.10
Vitamin premix ^1^	0.05	0.05
l-Lys·HCL (98.5%)	0.16	0.15
Mineral premix ^2^	0.50	0.38
Total	100.00	100.00
Calculated or analyzed nutrient content		
DE, Mcal/kg	3.22	3.16
^3^ Crude protein, %	15.37	14.22
^3^ Calcium, %	0.50	0.59
^3^ Total phosphorus, %	0.46	0.52
SID Lys, %	0.70	0.63

^1,2^ The premix provided the following vitamin and trace minerals per kilogram of diet: 4000 IU of vitamin A; 440 IU of vitamin E; 800 IU of vitamin D_3_; 0.5 mg of vitamin K; 1.0 mg of vitamin B_1_; 3.75 mg of vitamin B_2_; 1.0 mg of vitamin B_6_; 15 μg of vitamin B_12_; 12 mg of pantothenic acid; 10 mg of niacin; 1.3 mg of folic acid; 0.2 mg of d-biotin; Fe, 80 mg as ferrous sulfate; Zn, 100 mg as zinc sulfate; Cu, 6.6 mg as copper sulfate; Mn, 30 mg as manganese sulfate; I, 0.3 mg as potassium iodide; Se, 0.3 mg as sodium selenite. ^3^ Analyzed value.

**Table 2:** Mycotoxin levels (μg/kg) of each experimental diet and ingredient.

	Treatment								Ingredients				Maximum Allowed	
	Control	25% MC	50% MC	75% MC	100% MC	100% MW	100% MW + MC		NC	NW	MC	MW	China	EU
	Days 1–28 of experiment													
DON	203.5	289.4	329.2	442.4	532.7	227.2	604.8	DON	245.1	393.5	737.8	628.5	1000	900
ZEA	32.7	63.1	89.6	98.9	132.3	39.3	228.3	ZEA	44.6	35.2	185.3	106.4	100	250
AFB1	ND	2.4	10.4	8.1	8.9	4.8	12.4	AFB1	ND	ND	13.6	62.5	20	20
OA	ND	ND	ND	ND	ND	ND	ND	OA	ND	ND	ND	ND	100	50
FUM	ND	ND	ND	ND	ND	ND	ND	FUM	ND	ND	ND	ND	5000	5000
T-2	ND	ND	ND	ND	ND	ND	ND	T-2	ND	ND	ND	ND	500	-
	Day 29 to end of experiment													
DON	181.5	287.6	338.2	473.2	565.7	247.8	626.5							
ZEA	28.6	53.1	81.6	112.4	145.3	47.3	195.6							
AFB1	ND	2.8	5.4	6.8	8.4	6.6	10.9							
OA	ND	ND	ND	ND	ND	ND	ND							
FUM	ND	ND	ND	ND	ND	ND	ND							

DON, deoxynivalenol; ZEA, zearalenone; AFB1, aflatoxin B1; OA, ochratoxin; FUM, fumonisins; T-2, T-2 toxin; NC, normal corn; NW, normal wheat bran; MC, moldy corn; MW, moldy wheat bran; control, 0% moldy corn; 25% MC, 25% moldy corn; 50% MC, 50% moldy corn; 75% MC, 75% moldy corn; 100% MC, 100% moldy corn; 100% MW, 100 moldy wheat bran; 100% MW + MC, 100% MW and 100% MC. ND, not detected.

**Table 3:** Growth performances of gilts fed diets with different levels of moldy corn or wheat bran.

Treatment								SEM	*p* ^1^			*p* ^2^		
	Control	25% MC	50% MC	75% MC	100% MC	100% MW	100% MW+ MC		Overall	Linear of MC	Quadratic of MC	100% MC	100% MW	MC × MW
BW, kg														
1 day	48.43	48.49	48.35	48.48	48.55	48.78	48.51	1.30	ns	ns	ns	ns	ns	ns
28 days	69.00	69.44	68.27	67.09	67.43	68.12	65.72	1.82	ns	ns	ns	ns	ns	ns
56 days	93.20	90.09	88.16	88.76	88.51	87.39	85.72	2.20	ns	ns	ns	0.067	ns	ns
96 days	119.11	116.18	114.31	112.64	111.67	112.53	109.27	2.95	ns	0.056	ns	0.099	ns	ns
ADFI, kg/day														
1–28 days	1.97	2.07	2.06	1.94	2.19	1.97	1.87	0.07	ns	ns	ns	0.080	ns	0.086
29–56 days	2.77	2.77	2.78	2.74	2.77	2.57	2.78	0.10	0.098	ns	ns	ns	ns	ns
57–96 days	2.50	2.50	2.50	2.50	2.50	2.50	2.50	0.00	-	-	-	-	-	-
ADG, g/day														
1–28 days	734.7	748.3	711.4	664.6	674.3	690.6	614.7	39.8	ns	ns	ns	ns	*	ns
29–56 days	884.4 ^a^	737.3 ^b^	710.3 ^b^	774.0 ^ab^	752.8 ^ab^	688.1 ^b^	714.1 ^b^	43.6	*	0.073	*	*	ns	ns
57–96 days	647.8	652.2	653.9	597.1	578.9	632.8	588.7	46.9	ns	ns	ns	ns	ns	ns
F/G														
1–28 days	2.72	2.76	2.90	2.97	3.25	2.85	3.04	0.18	ns	0.075	ns	ns	ns	ns
29–56 days	3.20 ^a^	3.79 ^b^	3.92 ^b^	3.58 ^ab^	3.74 ^b^	3.85 ^b^	4.00 ^b^	0.17	*	ns	*	*	0.072	ns
57–96 days	4.17	4.00	3.99	4.54	4.52	4.09	4.43	0.40	ns	ns	ns	ns	ns	ns

Notes: ADFI, average daily feed intake; ADG, average daily gain; F/G, feed-to-gain ratio; MC, moldy corn; MW, moldy wheat bran; control, 0% moldy corn; 25% MC, 25% moldy corn; 50% MC, 50% moldy corn; 75% MC, 75% moldy corn; 100% MC, 100% moldy corn; 100% MW, 100 moldy wheat bran; 100% MW + MC, 100% MW, and 100% MC. Values are presented as the mean ± SEM. Within a row, values without the same lowercase superscript letters differ significantly (*p* < 0.05); *p*^1^, significance of linear and quadratic effects; *p*^2^, significance of effects of 100% MC, 100% MW, and their interaction. *, *p* < 0.05; ns, *p* > 0.1.

**Table 4:** Pubertal data of gilts fed diets with different levels of moldy corn or wheat bran.

Treatment								SEM	*p* ^1^			*p* ^2^		
	Control	25% MC	50% MC	75% MC	100% MC	100% MW	100% MW+ MC		Overall	Linear of MC	Quadratic of MC	100% MC	100% MW	MC × MW
Age at puberty, days	194.0	210.0	206.6	197.7	196.7	195.5	203.6	4.9	ns	ns	*	ns	ns	ns
BW at puberty, kg	115.2	116.6	111.9	113.3	106.7	114.9	111.4	4.0	ns	ns	ns	ns	ns	ns
BF at puberty, mm	12.1	12.1	13.1	11.3	11.3	10.3	11.3	0.6	0.089	ns	ns	ns	ns	ns
Cycle length, days	23.0	21.8	22.3	21.1	23.0	23.0	22.0	1.0	ns	ns	ns	ns	ns	ns

MC, moldy corn; MW, moldy wheat bran; control, 0% moldy corn; 25% MC, 25% moldy corn; 50% MC, 50% moldy corn; 75% MC, 75% moldy corn; 100% MC, 100% moldy corn; 100% MW, 100 moldy wheat bran; 100% MW + MC, 100% MW and 100% MC. Values are presented as the mean ± SEM. *p*^1^, significance of linear and quadratic effects; *p*^2^, significance of effects of 100% MC, 100% MW, and their interaction. *, *p* < 0.05; ns, *p* > 0.1; *n* = 6–7 per group.

**Table 5:** Organ development of gilts with different levels of moldy corn or wheat bran.

Treatment								SEM	*p* ^1^			*p* ^2^		
	Control	25% MC	50% MC	75% MC	100% MC	100% MW	100% MW + MC		Overall	Linear of MC	Quadratic of MC	100% MC	100% MW	MC × MW
BW at slaughter, kg	141.9	141.4	137.8	138.9	130.5	140.3	135.8	4.32	ns	*	0.062	0.071	ns	ns
Liver, g	1981.9	1992.4	2134.6	2051.92	1973.2	2117.9	2058.1	99.2	ns	ns	ns	ns	ns	ns
Liver, g/kg	14.1	13.9	15.4	15.4	15.1	15.1	15.3	0.9	ns	ns	ns	ns	ns	ns
Spleen, g	244.4	221.5	229.8	203.9	229.0	263.3	227.5	13.6	ns	ns	ns	ns	ns	ns
Spleen, g/kg	1.75	1.54	1.66	1.53	1.76	1.89	1.68	0.12	ns	ns	ns	ns	ns	ns
Kidney, g	379.7	389.0	360.7	382.3	350.2	399.5	382.7	19.1	ns	ns	ns	ns	ns	ns
Kidney, g/kg	2.7	2.7	2.6	2.8	2.7	2.9	2.8	0.16	ns	ns	ns	ns	ns	ns
Uterus, wt.	1114.0	967.4	913.2	871.7	1021.6	966.5	827.9	95.6	ns	ns	ns	ns	ns	ns
Uterus, g/kg	7.85	6.73	6.58	6.45	7.75	6.92	6.15	0.68	ns	ns	ns	ns	ns	ns
Ovary, g	13.4 ^a^	11.4 ^ab^	12.9 ^ab^	11.7 ^ab^	10.7 ^b^	12.2 ^ab^	9.8 ^b^	0.8	*	*	ns	**	ns	ns
Ovary, g/kg	0.096	0.079	0.093	0.087	0.082	0.087	0.073	0.007	ns	ns	ns	0.074	ns	ns
Ovarian follicle														
No. of small follicles, 1–3 mm	10.4	10.3	11.7	14.0	11.3	12.2	13.3	2.9	ns	ns	ns	ns	ns	ns
No. of large follicles, >3 mm	20.2 ^a^	14.3 ^ab^	14.0 ^ab^	11.8 ^ab^	12.5 ^ab^	12.7 ^ab^	7.5^b^	2.0	**	**	ns	*	*	ns
No. of follicles <1mm, *n*/cm^2^														
Primordial	8.9 ^a^	7.8 ^ab^	6.8 ^ab^	5.1 ^b^	4.9 ^b^	7.4 ^ab^	4.5 ^b^	0.9	**	**	ns	**	ns	ns
Growing	10.4 ^a^	7.3 ^ab^	7.5 ^ab^	6.0 ^b^	6.5 ^b^	8.3 ^ab^	6.0 ^b^	1.1	0.073	**	ns	*	ns	ns
Total primordial and growing	19.3 ^a^	15.2 ^a^	14.3 ^ab^	11.2 ^b^	11.3 ^b^	15.7 ^a^	10.5 ^b^	1.4	**	**	ns	**	ns	ns
No. of corpora lutea	20.8 ^a^	16.8 ^ab^	17.5 ^ab^	15.8 ^ab^	14.2 ^b^	18.2 ^ab^	13.7 ^b^	1.3	**	**	ns	**	ns	ns

MC, moldy corn; MW, moldy wheat bran; control, 0% moldy corn; 25% MC, 25% moldy corn; 50% MC, 50% moldy corn; 75% MC, 75% moldy corn; 100% MC, 100% moldy corn; 100% MW, 100 moldy wheat bran; 100% MW + MC, 100% MW and 100% MC. Values are presented as the mean ± SEM. Within a row, values without the same lowercase superscript letters differ significantly (*p* < 0.05); *p*^1^, significance of linear and quadratic effects; *p*^2^, significance of effects of 100% MC, 100% MW, and their interaction of both. *, *p* < 0.05; **, *p* < 0.01; *n* = 6–7 per group.

**Table 6:** Antioxidant status of gilts with different levels of moldy corn or wheat bran.

Treatment								SEM	*p* ^1^			*p* ^2^		
	Control	25% MC	50% MC	75% MC	100% MC	100% MW	100% MW+ MC		Overall	Linear of MC	Quadratic of MC	100% MC	100% MW	MC × MW
Serum														
MDA, nmol/mL	7.6 ^b^	9.6 ^b^	12.4 ^ab^	13.2 ^ab^	17.5 ^a^	7.8 ^b^	18.7 ^a^	2.47	**	**	ns	**	ns	ns
T-SOD, U/mL	43.9 ^a^	44.6 ^a^	39.3 ^ab^	36.8 ^ab^	34.9 ^ab^	40.4 ^ab^	30.1 ^b^	3.7	*	*	ns	*	ns	ns
Liver														
MDA, nmol/mg prot.	1.8 ^b^	2.0 ^ab^	2.1 ^ab^	2.2 ^ab^	2.3 ^ab^	2.1 ^ab^	2.4 ^a^	0.12	*	**	ns	**	0.053	ns
SOD, U/mg prot.	583.2 ^a^	499.2 ^ab^	564.5 ^ab^	566.5 ^ab^	421.5 ^b^	523.2 ^ab^	425.3 ^b^	28.5	**	**	*	**	ns	ns
Follicular fluid														
MDA, nmol/mL	3.3	4.3	5.4	5.4	6.3	5.5	7.4	1.1	ns	0.054	ns	0.055	ns	ns
T-SOD, U/mL	65.8 ^a^	66.0 ^a^	63.2 ^ab^	57.0 ^ab^	54.3 ^ab^	65.3 ^ab^	49.8 ^b^	3.9	*	*	ns	*	ns	ns

MC, moldy corn; MW, moldy wheat bran; control, 0% moldy corn; 25% MC, 25% moldy corn; 50% MC, 50% moldy corn; 75% MC, 75% moldy corn; 100% MC, 100% moldy corn; 100% MW, 100 moldy wheat bran; 100% MW + MC, 100% MW and 100% MC. Values are presented as the mean ± SEM. Within a row, values without the same lowercase superscript letters differ significantly (*p* < 0.05); *p*^1^, significance of linear and quadratic effects; *p*^2^, significance of effects of 100% MC, 100% MW, and their interaction. *, *p* < 0.05; **, *p* < 0.01; *n* = 6–7 per group.

**Table 7:** Hormone secretions of gilts with different levels of moldy corn or wheat bran.

Treatment								SEM	*p* ^1^			*p* ^2^		
	Control	25% MC	50% MC	75% MC	100% MC	100% MW	100% MW + MC		Overall	Linear of MC	Quadratic of MC	100% MC	100% MW	MC × MW
Serum FSH														
0 min	9.07	9.40	10.35	10.71	10.39	10.09	10.22	1.52	ns	ns	ns	ns	ns	ns
60 min	11.66	10.14	10.29	10.52	10.75	10.07	10.94	1.45	ns	ns	ns	ns	ns	ns
120 min	9.98	9.98	10.76	9.68	10.65	9.86	9.27	1.29	ns	ns	ns	ns	ns	ns
Serum LH														
0 min	3.49	3.28	3.29	3.42	3.40	3.36	3.04	0.69	ns	ns	ns	ns	ns	ns
60 min	2.89	2.91	3.12	3.26	3.51	3.39	2.70	0.65	ns	ns	ns	ns	ns	ns
120 min	3.03	3.29	3.19	3.56	2.89	3.64	3.25	0.63	ns	ns	ns	ns	ns	ns
Serum IGF-1, ng/mL	169.9 ^a^	101.7 ^bc^	100.4 ^bc^	120.1 ^b^	109.4 ^bc^	104.5 ^b^	90.3 ^c^	6.34	**	ns	ns	**	**	**
Serum E_2_, pg/mL	147.4	146.8	106.2	120.5	128.9	113.5	113.5	15.9	ns	ns	ns	ns	ns	ns
Serum AMH,	15.93 ^a^	13.63 ^ab^	13.74 ^ab^	11.46 ^b^	11.13 ^b^	13.59 ^ab^	11.0 ^b^	1.04	**	**	ns	**	ns	ns
Follicular fluid E_2_, ng/mL	194.8 ^a^	136.5 ^bc^	150.2 ^b^	124.5 ^bc^	148.2 ^bc^	130.3 ^bc^	105.5 ^c^	9.9	**	**	**	**	**	**

Notes: MC, moldy corn; MW, moldy wheat bran; control, 0% moldy corn; 25% MC, 25% moldy corn; 50% MC, 50% moldy corn; 75% MC, 75% moldy corn; 100% MC, 100% moldy corn; 100% MW, 100 moldy wheat bran; 100% MW + MC, 100% MW and 100% MC. Values are presented as the mean ± SEM. Within a row, values without the same lowercase superscript letters differ significantly (*p* < 0.05); *p*^1^, significance of linear and quadratic effects; *p*^2^, significance of effects of 100% MC, 100% MW, and their interaction. **, *p* < 0.01; *n* = 6–7 per group.

## Data Availability

Not applicable.
